# The College of Nursing (Limited by Guarantee)

**Published:** 1920-11-13

**Authors:** 


### The College of Nursing (Limited by Guarantee).

Members are reminded of the Extraordinary General Meeting to be held on Saturday afternoon, November 20, at 3 p.m., at the Royal Society of Medicine, to confirm the Resolutions passed at the meeting of November 4, 1920.

### UNEMPLOYMENT INSURANCE ACT.

At the close of the meeting of members of the College on Saturday, November 20, at the Royal Society of Medicine, an expert will explain the above Act as it affects the nursing profession.

Discussion will be invited. It is thought many members will wish to avail themselves of this opportunity of becoming familiar with the details and administration of the Act. The meeting will commence at 3.30 p.m.

### COMMISSIONED RANK FOR SERVICE NURSES.

The Local Centres of the College of Nursing are sending in to headquarters their opinions on the above subject.

It would aid the Committee which is considering the question to receive the opinions of Regulars in the Queen Alexandra's Imperial Military Nursing Service and in the Royal Naval Nursing Service. Letters should be addressed to the Secretary, The College of Nursing, Limited, 7 Henrietta Street, Cavendish Square, London, W. 1.

### NOTICE TO MEMBERS.

Mr. Archer Ryland, F.R.C.S. (Ed.), M.R.C.S., F.R.C.P. (Lond.), 50 Harley Street, W. 1, has offered his services as honorary aurist and laryngologist to members of the College living in London. The Council has gratefully accepted Mr. Ryland's kind offer. Members wishing to avail themselves of Mr. Ryland's professional services must first notify the Secretary.

